# Supplementary material for: Patterns of Intron Gain and Loss in Fungi
Source: PLoS Biol. 2004 Nov 30;2(12):e422. doi: 10.1371/journal.pbio.0020422 (PMC532390; doi:10.1371/journal.pbio.0020422)
Supplement: Table S1 — Also available at http://genes.mit.edu/NielsenEtAl/. (4.3 MB ZIP). [file pbio.0020422.st001.zip › NielsenEtAl/html/471.html]

AN8041.1.NCU01528.1.MG01084.1.FG06257.1


```
 CLUSTAL W (1.82) Multiple Sequence Alignments - Introns Inserted


Sequence 1: MG01084.1	336 aa
Sequence 2: FG06257.1	338 aa
Sequence 3: NCU01528.1	338 aa
Sequence 4: AN8041.1	239 aa
Alignment Length: 339 aa
Number Identitical Residues: 167 aa
Alignment Score (without introns) 8536


MG01084.1 	--MVK~CGINGFGRIGRI~VFRNA~IEHPDCEIVAVNDPFIEPKYA0KYMLEYDSTHGRF
NCU01528.1	-MVVK~VGINGFGRIGRI~VFRNA~IEHDDIHIVAVNDPFIEPKYA0AYMLRYDTTHGNF
FG06257.1 	MAPIK~VGINGFGRIGRI~VFRNA~VEHPDIEVVAVNDPFIEPHYA0VYMLKYDSSHGIF
AN8041.1  	-MAPK0VGINGFGRIGRI0VFRNA2IEAGTVDVVAVNDPFIETHYA0AYMLKYDSQHGQF
          	    *  *********** ***** :*    .:*********.:**  ***.**: ** *

MG01084.1 	KGTVEVSGSDLVVNGKKVKFYTERDPANIPWSETGAEYVVESTGVFTTTDKASAHLKGGA
NCU01528.1	KGTIEVDGADLVVNGKKVKFYTERDPAAIPWSETGADYIVESTGVFTTTEKASAHLKGGA
FG06257.1 	KGEVGNQGNDLVVNGKTIKFYSERDPANIKWSETGADYVVESTGVFTTIDKAKAHLQGGA
AN8041.1  	KGTIETYDEGLIVNGKKIRFHTERDPANIPWGQDGAEYIVESTGVFTTQEKASAHLKGGA
          	** :   . .*:****.::*::***** * *.: **:*:********* :**.***:***

MG01084.1 	KKVIISAPSADAPMYVMGVNEKSYDGSASVISNASCTTNCLAPLAKVINDKFGIVEGLMT
NCU01528.1	KKVIISAPSADAPMYVMGVNNETYDGSADVISNASCTTNCLAPLAKVIHDNFTIVEGLMT
FG06257.1 	KKVIISAPSADAPMYVVGVNENKYDGSADIISNASCTTNCLAPLAKVINDKFGIVEGLMT
AN8041.1  	KKVVISAPSADAPMFVMGVNNETYKKDIQVLSNASCTTNCLAPLAKVINDNFGIIEGLMT
          	***:**********:*:***::.*. . .::*****************:*:* *:*****

MG01084.1 	TVHSYTATQKTVDGPSAKDWRGGRGAAQNIIPSSTGAAKAVGKVIPALNGKLTGMSMR~V
NCU01528.1	TVHSYTATQKTVDGPSAKDWRGGRTAAQNIIPSSTGAAKAVGKVIPDLNGKLTGMAMR~V
FG06257.1 	TVHSYTATQKTVDGPSSKDWRGGRGAAQNIIPSSTGAAKAVGKVIPELNGKLTGMSMR~V
AN8041.1  	TVHSYTATQKVVDGPSAKDWRGGRTAATNIIPSSTGAAKAVGKVIPSLNGKLTGMAMR1C
          	**********.*****:******* ** ****************** ********:**  

MG01084.1 	PTANVSVVDLTCRLEKGASYEEIKAAIKEAADGPLKG1ILEYTEDDVVSSDMIGNNASSI
NCU01528.1	PTANVSVVDLTARIEKGATYDEIKEVIKKASEGPLAG~ILAYTEDEVVSSDMNGNPASSI
FG06257.1 	PTANVSVVDLTVRLEKGASYDQIKQVIKEASEGDLKG~VLAYTEDDVVSSDLNGNTNSSI
AN8041.1  	RQEGF--------------------------------~----------------------
          	   ..                                                       

MG01084.1 	FDAQAGIALNDKFVKLVSWYDNEWGYSRRVIDLVTYISKVDGGK-
NCU01528.1	FDAKAGISLNKNFVKLVSWYDNEWGYSRRVLDLISYISKVDAKKA
FG06257.1 	FDAKAGISLNDNFVKLVSWYDNEWGYSRRVLDLLAHVAKVDASK-
AN8041.1  	---------------------------------------------
          	
```
